# Unveiling the microbial communities and metabolic pathways of *Keem*, a traditional starter culture, through whole-genome sequencing

**DOI:** 10.1038/s41598-024-53350-3

**Published:** 2024-02-18

**Authors:** Babita Rana, Renu Chandola, Pankaj Sanwal, Gopal Krishna Joshi

**Affiliations:** 1 Department of Biotechnology, School of Life Sciences, Hemvati Nandan Bahuguna Garhwal University, Srinagar Garhwal, Uttarakhand India; 2 Department of Biochemical Engineering, BTKIT, Dwarahat, Uttarakhand India

**Keywords:** Biotechnology, Microbiology

## Abstract

Traditional alcoholic beverages have played a significant role in the cultural, social, and culinary fabric of societies worldwide for centuries. Studying the microbial community structure and their metabolic potential in such beverages is necessary to define product quality, safety, and consistency, as well as to explore associated biotechnological applications. In the present investigation, Illumina-based (MiSeq system) whole-genome shotgun sequencing was utilized to characterize the microbial diversity and conduct predictive gene function analysis of *keem*, a starter culture employed by the Jaunsari tribal community in India for producing various traditional alcoholic beverages. A total of 8,665,213 sequences, with an average base length of 151 bps, were analyzed using MG-RAST. The analysis revealed the dominance of bacteria (95.81%), followed by eukaryotes (4.11%), archaea (0.05%), and viruses (0.03%). At the phylum level, Actinobacteria (81.18%) was the most abundant, followed by Firmicutes (10.56%), Proteobacteria (4.00%), and Ascomycota (3.02%). The most predominant genera were *Saccharopolyspora* (36.31%), followed by *Brevibacterium* (15.49%), *Streptomyces* (9.52%), *Staphylococcus* (8.75%), *Bacillus* (4.59%), and *Brachybacterium* (3.42%). At the species level, the bacterial, fungal, and viral populations of the *keem* sample could be categorized into 3347, 57, and 106 species, respectively. Various functional attributes to the sequenced data were assigned using Cluster of Orthologous Groups (COG), Non-supervised Orthologous Groups (NOG), subsystem, and KEGG Orthology (KO) annotations. The most prevalent metabolic pathways included carbohydrate, lipid, and amino acid metabolism, as well as the biosynthesis of glycans, secondary metabolites, and xenobiotic biodegradation. Given the rich microbial diversity and its associated metabolic potential, investigating the transition of *keem* from a traditional starter culture to an industrial one presents a compelling avenue for future research.

## Introduction

Fermentation is an age-old art practiced since time immemorial by mankind to prepare a variety of food items and alcoholic beverages. However, the occurrence and role of microbes in the fermentation process were not discovered until the last 200 years. Besides its role in producing various commodity chemicals, solvents, and antibiotics, fermentation is also widely employed in the creation of diverse foodstuffs and alcoholic beverages^1^. Fermented foods are edibles or beverages made through the enzymatic conversion of substrates by allowing controlled microbial growth. During the process, the chemical composition of raw materials changes, leading to the development of a product with unique and improved taste, flavour, and nutritional quality^2^. Alcoholic beverages are unique fermentation products prepared and utilized all over the world for drinking, entertainment, as well as for several customary and cultural practices^3^. They are one of the most sellable products of fermentation and a great source of revenue for governments. The global alcoholic beverages market size was $508.87 billion in 2021 and is expected to grow to $802.02 billion in 2026 at a CAGR of 9.4%^4^. Although wine-making has been industrialized globally for a long time, the art is still being practiced in several places, particularly in tribal regions, as a part of an age-old tradition and culture. The microbiology of traditional alcoholic beverages made in such places is still poorly understood^5^.

During alcohol fermentation, a wide range of microorganisms are initially present but later yeast and lactic acid bacteria predominate to carry out the process and provide exclusive taste and flavor to the end product^6^. Starter culture or inoculum is the source of desired microorganisms for alcoholic fermentation. It is believed that China is the first country that initiated the use of starter inoculum in the fermentation process and then spread to nearby neighboring Asian countries^7^. There are different local names for such starter cultures such as *Chu* (China), *Ragi-tapai* (Malaysia), *Bubod* (Philippines), *Nuruk* (Korea), *Ragi* (Indonesia) and *Marcha* (India) etc^8^. The starter culture is a mixture of some medicinal herbs and desired microbiota such as molds/fungi, yeast & bacteria required to carry out the fermentation process using different substrates^6^. The beneficial microorganisms present in the starter cultures are primarily responsible for carbohydrate and protein metabolism. They are also known to produce diverse enzymes, flavoring compounds, vitamins, and other valuable constituents while suppressing the growth of pathogenic microbes^9^. Therefore, this is of great importance to determine the microbial community structure and metabolic interactions in the starter culture to develop a comprehensive understanding of the fermentation process as well as bioprospecting predictions.

Microbial diversity estimation by traditional culture-dependent methods has great limitations as a very less number of microbes originally present in an environment can be cultivated by it^10,11^. Metagenomics offers a promising alternative to it as it provides access to almost all the microbes present in an environment by way of analyzing genomic DNA extracted directly from a sample^12^. The scope of metagenomics has been widened further by the rapid advancements in the field of high throughput sequencing (HTS). Thanh et al. (2008)^13^ studied the microbiota of *banh men*, a starter used by Vietnamese to prepare traditional alcohol, through Polymerase Chain Reaction (PCR)-based Denaturing Gradient Gel Electrophoresis (DGGE). Shah et al. (2017)^14^ explored the dominance of proteobacteria in the amylolytic starter inoculum *Marcha* and *Thiat*, prepared by tribal people of Sikkim and Meghalaya state in India, by targeted amplicon sequencing method. The community structure and metabolic potential of microorganisms present in traditional starter cultures have been investigated through whole metagenome shotgun sequencing in the recent past^6,15^.

Jaunsar-Bawar is a tribal area located in the hilly region of the state of Uttarakhand, India. In this region, traditional distilled and undistilled alcoholic beverages are produced using a common inoculum locally called *keem*. Native villagers prepare this starter inoculum during the rainy season by using locally available herbs and medicinal plants. These herbs and plant parts are crushed into a fine powder and mixed with barley flour (5:1). The resulting mixture is kneaded into a dough, from which cakes weighing around 2 kg each are prepared. These cakes are kept in the dark for approximately 25–30 days, and then they are sun-dried or air dried before use^16^. In a study by Bhardwaj et al. (2015)^17^, four yeast strains and five lactic acid bacterial strains in *keem* were characterized through culture-dependent microbiological analysis. However, the community structure, as well as the metabolic capabilities of the microbial population present in the starter culture, remain unknown. Determining the microbial diversity associated with *keem* and its metabolic potential will play a crucial role in determining the characteristics of the end products, such as ethanol, organic acids, higher alcohols, and ester derivatives, among others. While this tribe is one of the most populated among all tribes in Uttarakhand, they still adhere to their customs and ethnic practices. However, the local people are not well-informed about the scientific basis of their traditional art. Therefore, studying and documenting the scientific principles behind their traditional foods, beverages, and starter inoculum is of utmost importance. This study represents the first comprehensive analysis of the bacterial, fungal, and archaeal communities as well as metabolic pathways associated with *keem* through shotgun whole genome sequencing. We have also compared the microbiome of *keem* with other stater cultures studied by earlier workers.

## Results

### Overview of whole-metagenome sequence statistics

The whole genome shotgun sequencing resulted in 22,350,940 sequences having 3,374,991,940 base pairs with an average length of 151 bps. Among the tested sequences, 13,685,727 sequences that belonged to human and plant origin were not considered to qualify the QC pipeline. Among the QC passed sequences, 48,988 (1%) belonged to ribosomal RNA genes, 5,144,498 (65.48%) contained predicted proteins with known functions, and 2,662,938 (33.90%) related to predicted proteins with unknown function. On the other hand, 808,789 sequences (3.62%) remained unknown and could not be ascribed to any function. A more detailed sequence analysis statistics of *keem* metagenome is also given (Supplementary Data Table S1).

### Functional category hits distribution of *keem* metagenome

**Figure 1:**
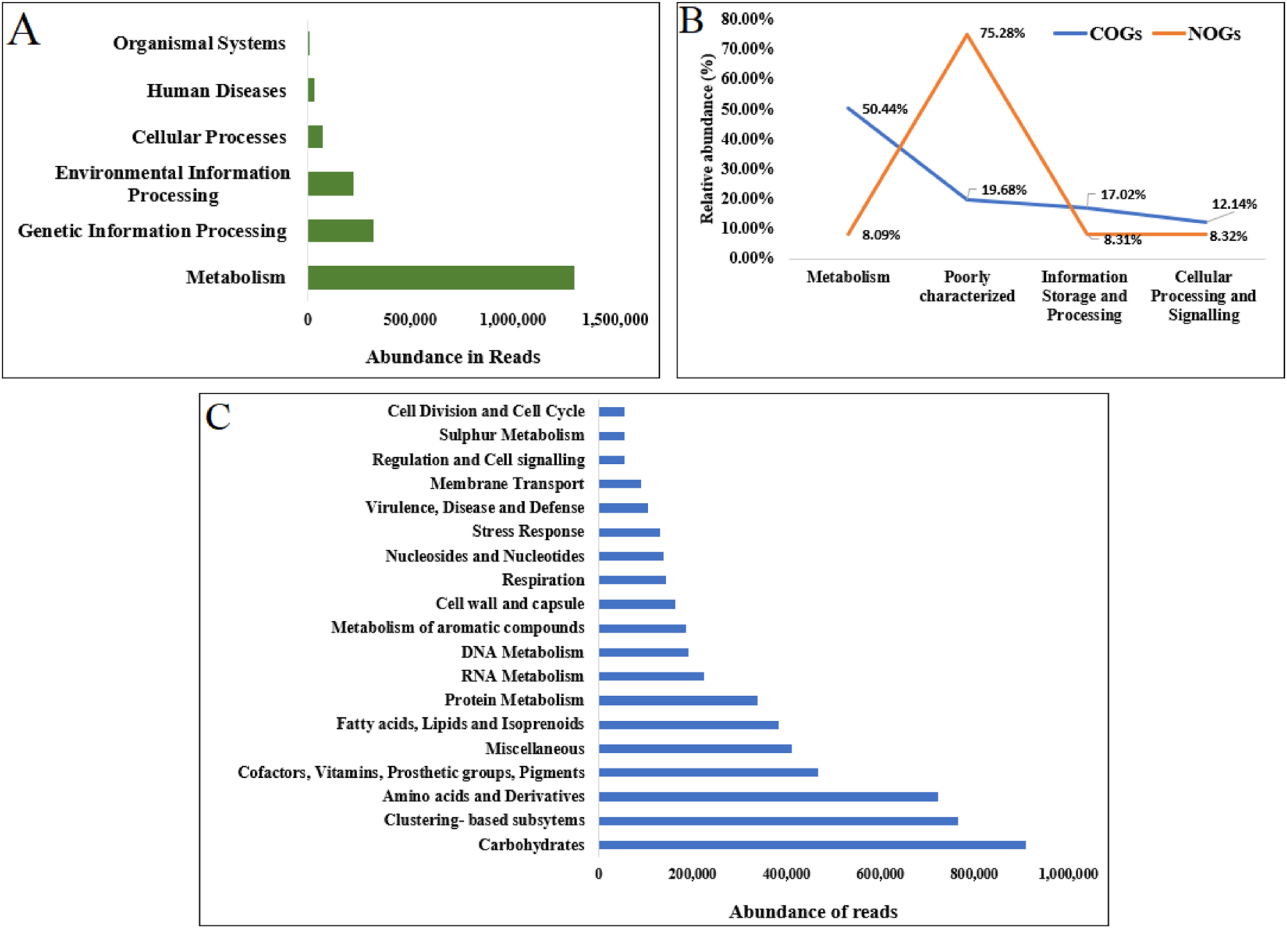
Illustration of read abundance with different functional hit category. (**A**) KEGG annotation (**B**) COGs and NOGs annotation and (**C**) Sub-system annotation.

The KO or KEEG Orthology annotation described 66.73% of total sequences attributing to metabolism; 16.42% to genetic information processing; 11% to environmental information processing; 3.53% to cellular processes; 1.57% to human diseases and 0.24% to organismal systems (Fig. 1A). The Cluster of Orthologous Groups (COGs) annotation resulted in the identification of 1,331,157 (50.44%) sequences belonging to metabolism; 449,062 (17.02%) related to information storage and processing and 339,513 (12.14%) coding for cellular processes and signaling. A total number of 519,330 (19.68%) sequences remained poorly characterized (Fig. 1B). In NOG annotation, a large proportion i.e., 117,362 (75.28%) sequences remained poorly characterized. Of the remaining sequences, 12,964 (8.32%) were assigned to cellular processes and signaling, 12,952 (8.31%) to information storage and processing, and 12,615 (8.09%) to metabolism (Fig. 1B). The Subsystem annotation identified 18.80% sequences for carbohydrates; 13.29% for clustering-based subsystems; 12.56% for amino acids and derivatives; 8% for cofactors, vitamins, prosthetic groups, and pigments; 7.15% for miscellaneous function; 6.66% for fatty acids, lipids and isoprenoids; 5.89% for protein metabolism; 3.90% for RNA metabolism; 3.32% for DNA metabolism, 3.22% for the metabolism of aromatic compounds; 2.83% for cell wall and capsule; 2.49% for respiration; 2.40% for nucleosides and nucleotides (Fig. 1C).

### Metabolic pathways detected in *keem*

**Figure 2:**
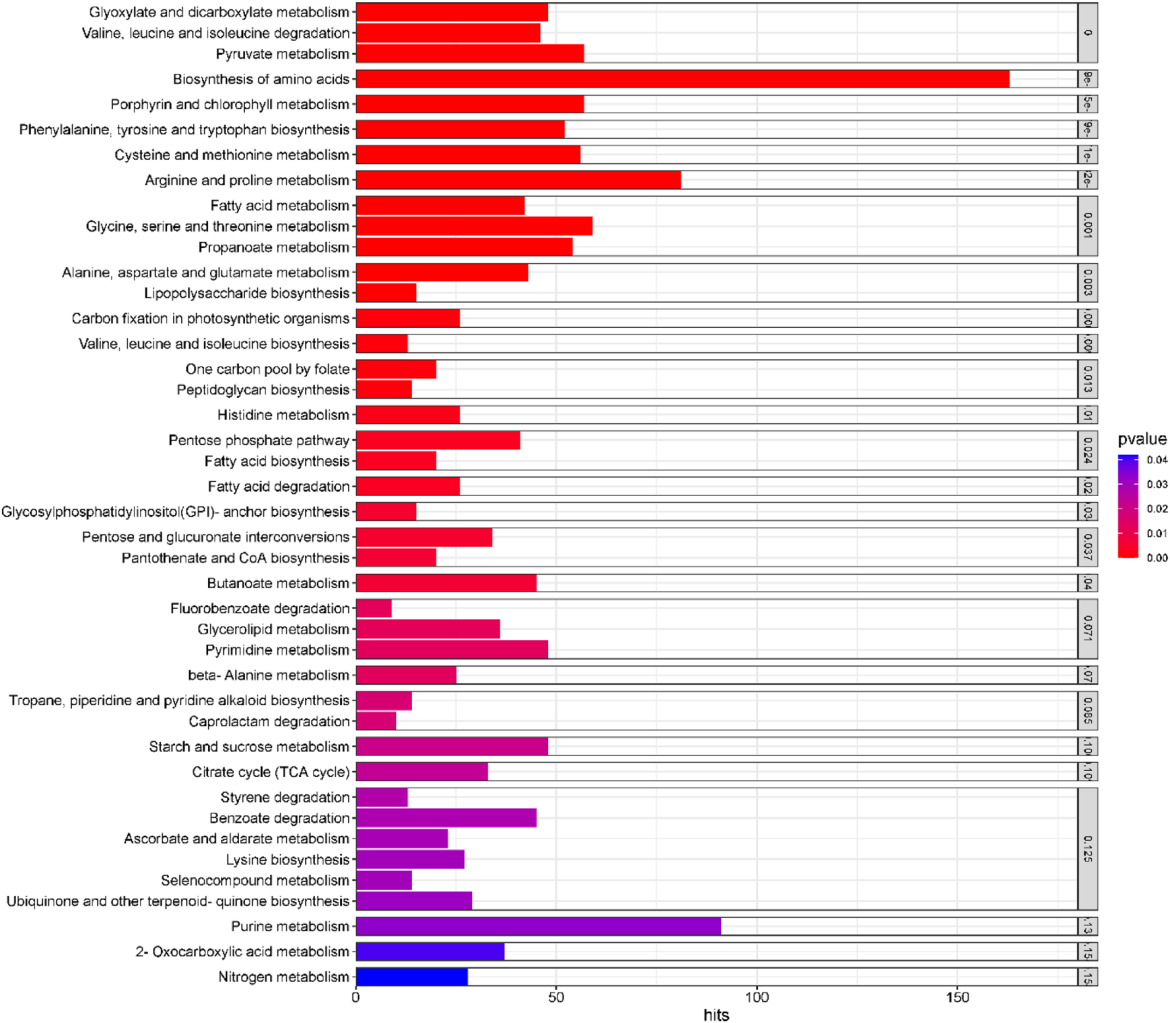
KEGG pathway analysis from shotgun sequence data of *keem* through SRplot.

A total number of 42 metabolic pathways harboring a varying number of genes in a statistically significant manner were detected in *keem* metagenome (Supplementary Data Table S2). Prevalent metabolic pathways include carbohydrate metabolism, lipid metabolism, amino acid metabolism, metabolism of amino acids, glycan biosynthesis & metabolism, biosynthesis of other secondary metabolites, xenobiotic biodegradation metabolism, metabolism of cofactors & vitamins, nucleotide metabolism, and energy metabolism. Based on the *p*-value of the genes responsible for each identified pathway, the KEGG pathway map was created using freely accessible web server SRplot (Fig. 2).

### Taxonomic hit distribution

**Figure 3:**
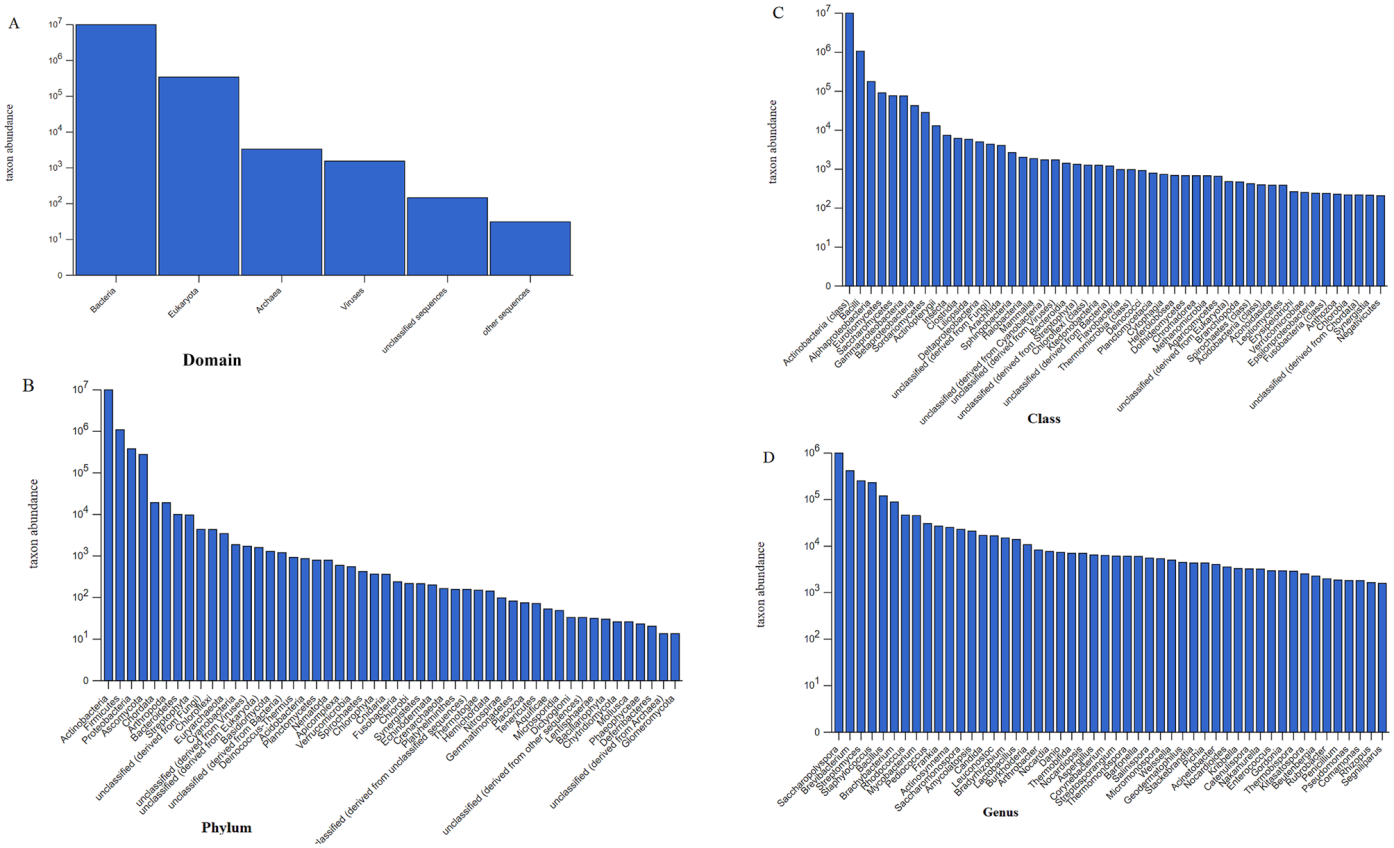
The most abundant population at different taxonomic levels in the *keem* sample (**A**) domain, (**B**) phylum, (**C**) class and (**D**) genus level.

The MG-RAST analysis of the sequence reads revealed the abundance of bacteria (95.81%) over Eukaryota (4.00%), archaea, and others (0.08%) in the test starter culture (Fig. 3A). Among all the microbes present in the starter sample, Actinobacteria was the most dominant phylum (81.18%) followed by Firmicutes (10.56%) and Proteobacteria (4.00%); all falling within the domain bacteria. With a representation of 3.02% of the total sequences, Ascomycota was the most abundant phylum detected within the domain of fungi (Fig. 3B). The members under the group of algae, chordates, arthropods and viruses were also represented by the sequence data albeit at a low proportion compared to the above-mentioned phyla.

The class level distribution shows the richness of Actinobacteria (class) represented by 82.38% of total sequences followed by Bacilli (10.48%), Alphaproteobacteria (2.01%) and Eurotiomycetes (1.09%) (Fig. 3C). Whereas at genus level, 36.31% of total sequences belonged to *Saccharopolyspora*, 15.49% to *Brevibacterium*, and 9.52% to *Streptomyces*. The other prominent genera were *Staphylococcus* (8.75%), *Bacillus* (4.59%), and *Brachybacterium* (3.42%) (Fig. 3D).

### Microbial diversity analysis

MG-RAST analysis of the whole genome sequences as obtained from the *keem*, revealed an alpha-diversity of 123 species. The rarefaction curve of annotated species richness is a function of the number of sequences sampled in the present study (Supplementary Data Figure S1).

### Bacteria

**Figure 4:**
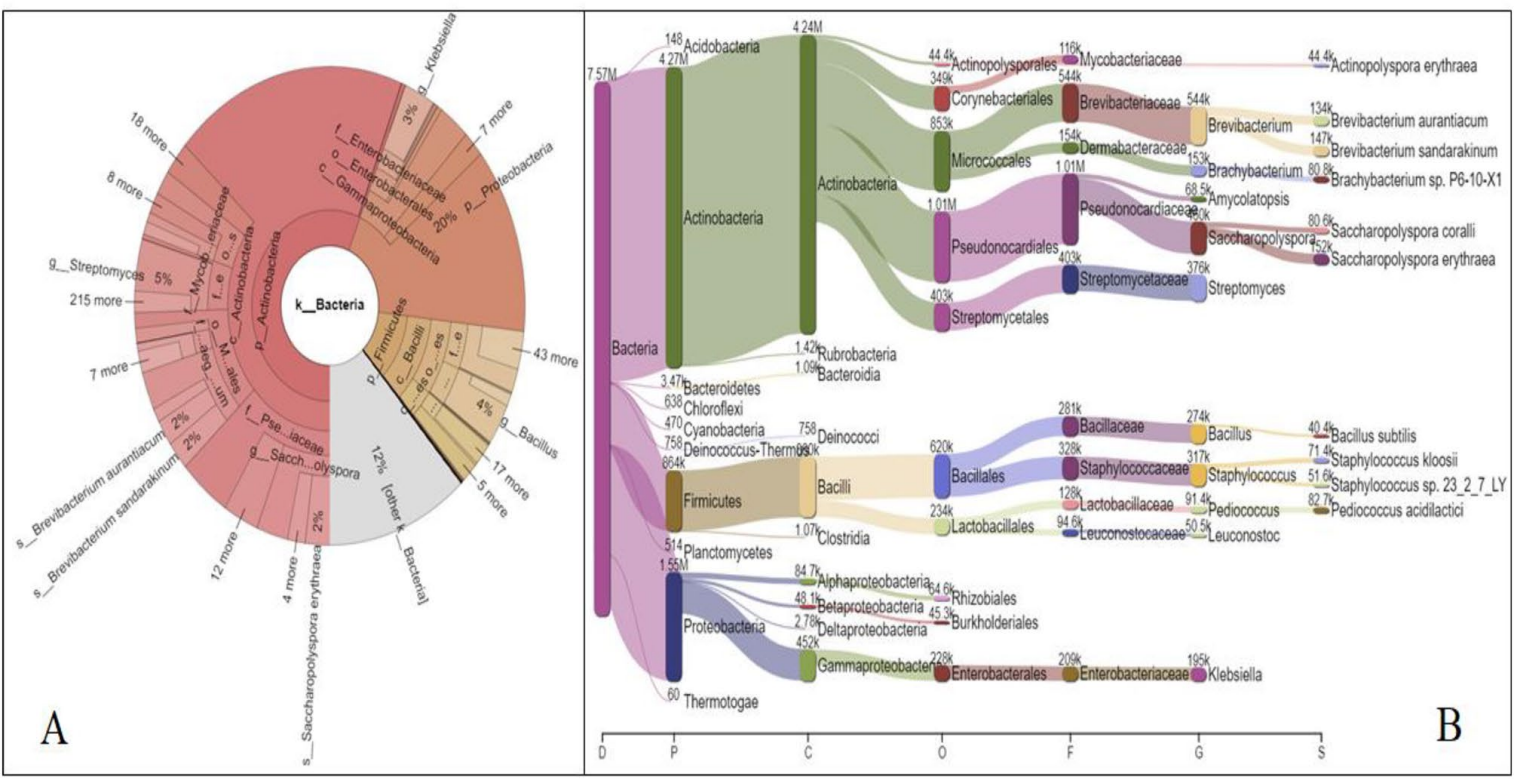
Krona plot (**A**) and Sankey visualization (**B**) of bacterial diversity identified in *keem*.

The sequence data analysis through Pavian R package v1.0.0 resulted in the identification of a total number of 3347 bacterial species. The top ten bacterial species in the decreasing order of abundance in the test sample were *Saccharopolyspora erythraea* (152,138 sequences), *Brevibacterium sandarakinum* (146,766 sequences), *B. aurantiacum* (134,008 sequences); *Brachybacterium* sp. P6-10-X1(80,772 sequences); *Saccharopolyspora coralli* (80,580 sequences); *Staphylococcus kloosii* (71,386); *Staphylococcus sp. 23_2_7_LY* (51,556 sequences)*; Actinopolyspora erythraea (*44,356 sequences); *Bacillus subtilis* (40,412 sequences); *Bradyrhizobium* sp. BTAi1(30,622 sequences). A comprehensive detail of the bacterial diversity of *keem* have been represented though Krona plot and Sankey visualization (Fig. 4A, B).

### Lactic acid bacteria

The Lactic Acid Bacteria present in the *keem* sample belonged to 6 genera distributed in 12 species. In the decreasing order of abundance these were *Pediococcus acidilactici, Weissella cibaria, Leuconostoc pseudomesenteroides, Leuconostoc mesenteroides, Weissella confusa, Pediococcus pentosaceus, Limosilactobacillus reuteri, Lactiplantibacillus plantarum, Weissella paramesenteroides, Leuconostoc suionicum, Lactobacillus johnsonii* and *Leuconostoc citreum.*

### Archaea

**Figure 5:**
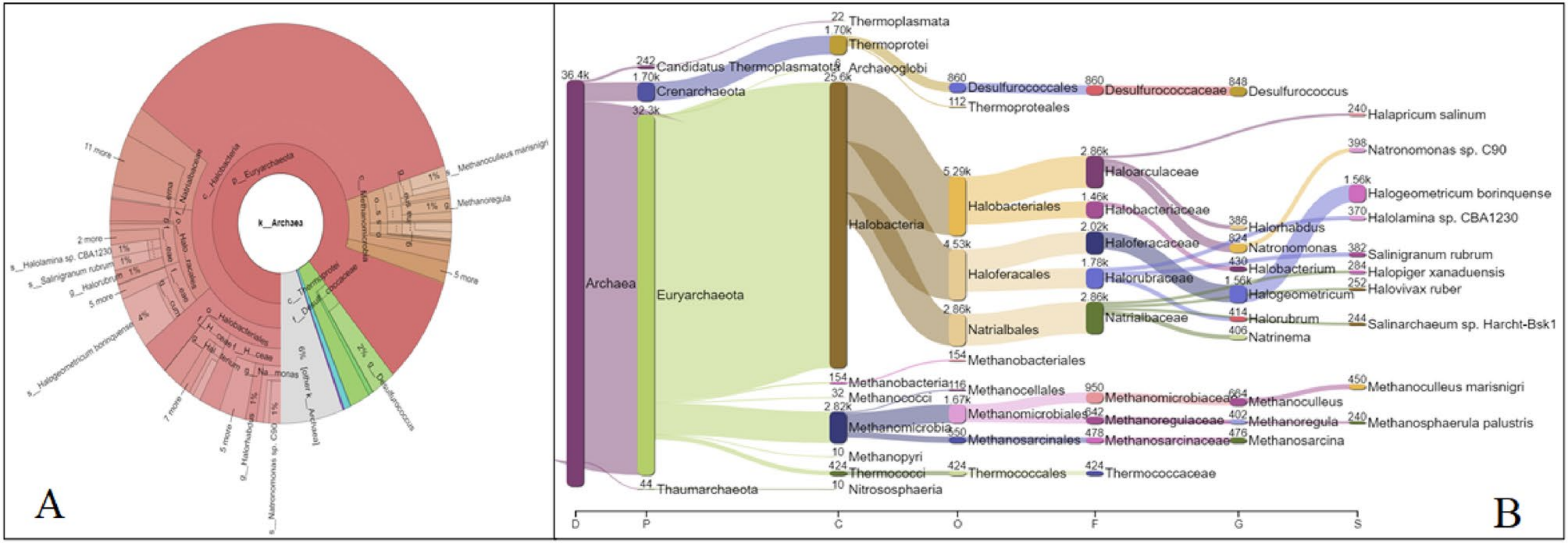
Krona plot (**A**) and Sankey visualization (**B**) of archaeal diversity identified in *keem*.

The sequence data analysis resulted in the identification of 174 archaea classified at different taxonomic level. The most prominent phylum was Euryarchaeota followed by Crenarchaeota, Candidatus and Thaumarchaeota. The Sankey visualization and Krona plot representing the cumulative and comparative taxonomical classification of archaeal population of *keem* sample is shown in Fig. 5A, B.

### Fungi

**Figure 6:**
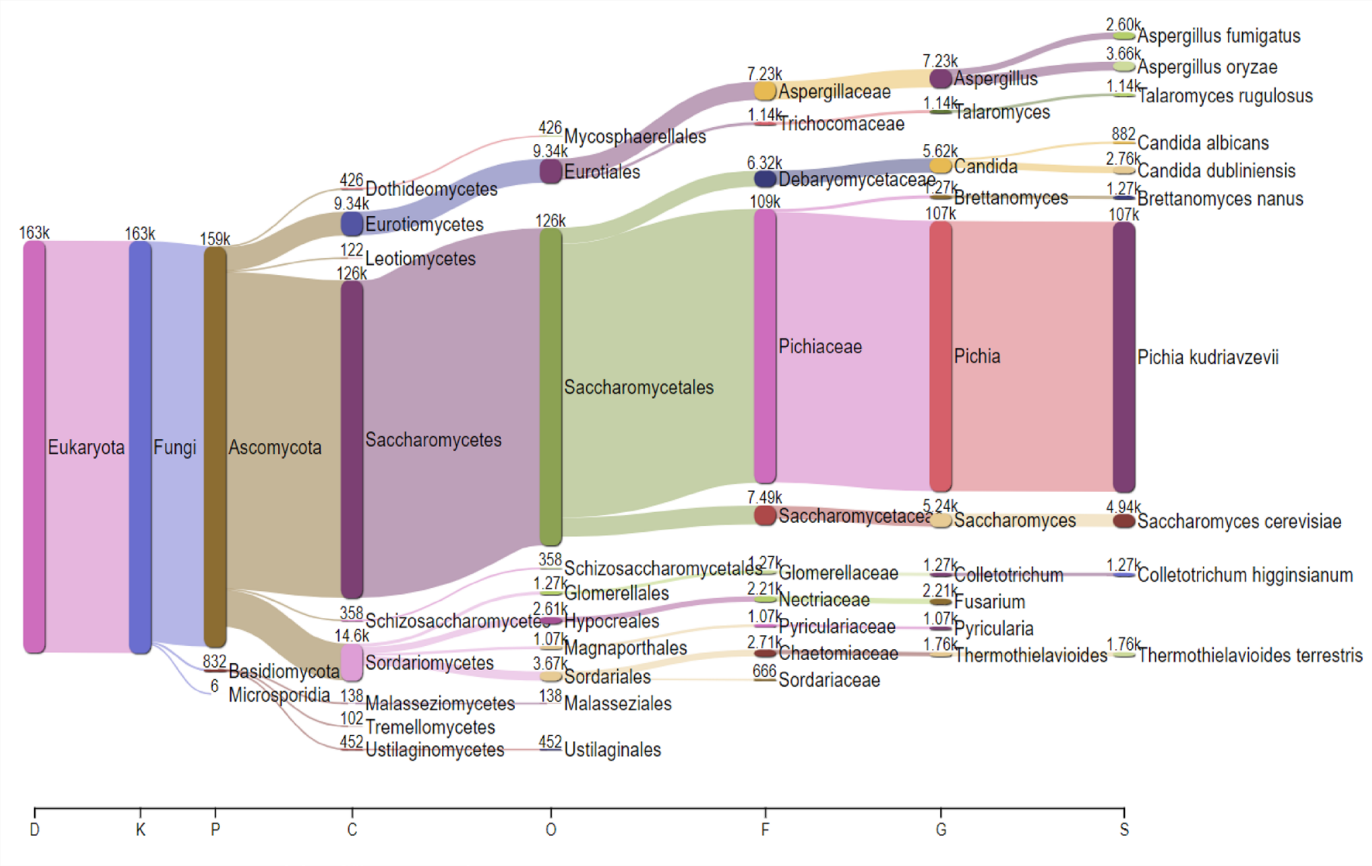
Sankey plot/visualization showing the taxonomic distribution of fungal diversity found in *keem*.

MG-RAST analysis of sequence data led to the identification of 57 fungal species in the test sample. The ten most abundant species are *Aspergillus oryzae, Aspergillus fumigatus, Thermothielavioides terrestris, Colletotrichum higginsianum, Neurospora crassa, Thermothelomyces thermophilus, Sugiyamaella lignohabitans, Fusarium pseudograminearum, Sporisorium graminicola and Pochonia chlamydosporia.* A more comprehensive visualization of fungal diversity of *keem* sample is given in Fig. 6.

### Yeast

The yeast population distributed in 18 genera and 29 species were identified in the starter culture studied in the present investigation. The names are listed in descending order of their relative richness in the test metagenome, namely *Pichia kudriavzevii; Saccharomyces cerevisiae, S. paradoxus* & *S. eubayanus; Candida dubliniensis, C. albicans, C. orthopsilosis* & *C. glabrata; Brettanomyces nanus; Cryptosporidium parvum; Schizosaccharomyces pombe; Debaryomyces hansenii; Scheffersomyces stipites; Lachancea thermotolerans; Naumovozyma dairenensis* & *N. castellii; Kluyveromyces marxianus* & *K. lactis; Ogataea parapolymorpha; Kazachstania africana & K. naganishii; Zygosaccharomyces rouxii; Torulaspora delbrueckii & T. globose; Cryptococcus neoformans & C. gattii* VGI*; Eremothecium gossypii, E. cymbalariae* & *E. sinecaudum*.

### Virus

**Figure 7:**
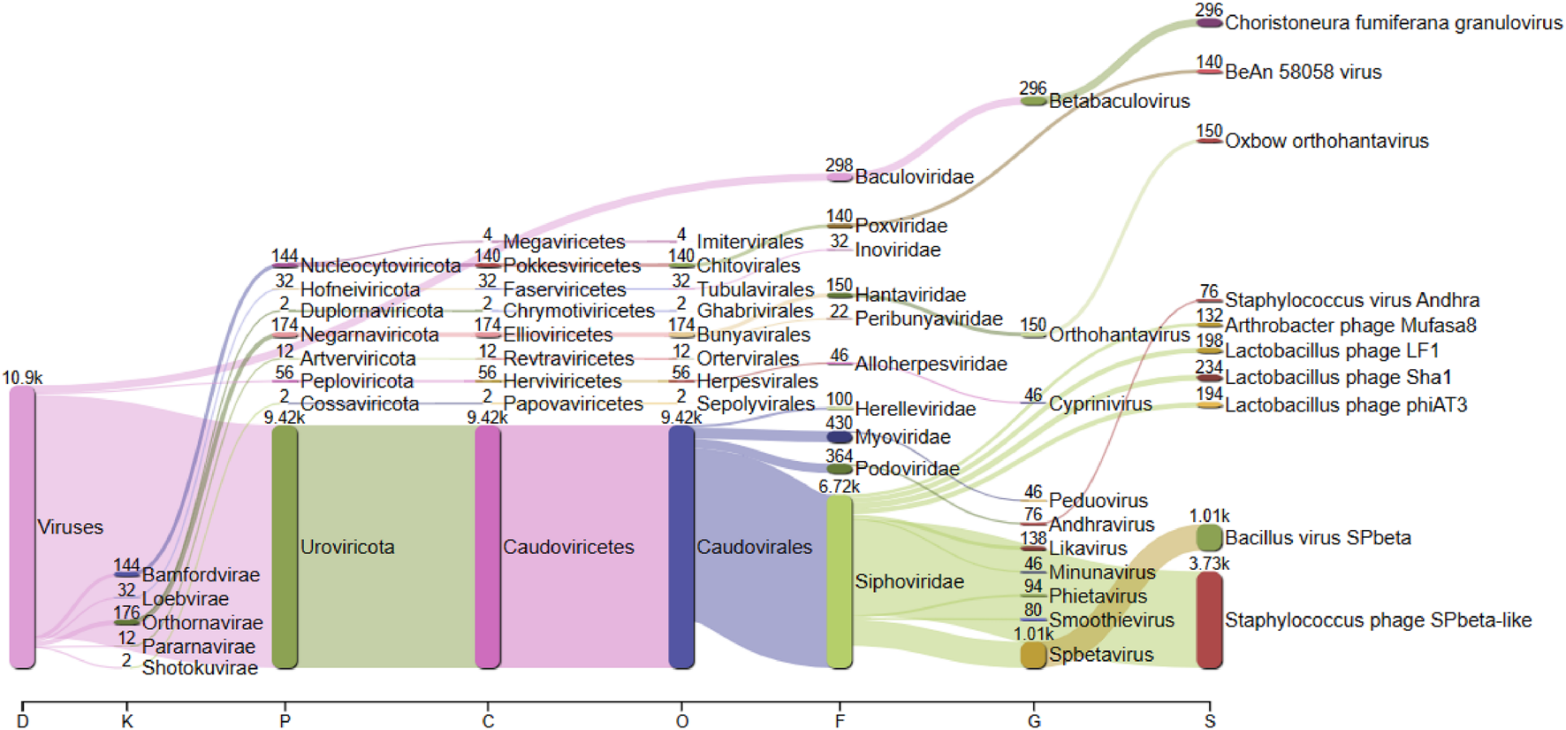
Sankey plot/visualization showing the taxonomic distribution of viral diversity found in *keem*.

The viral population of *keem* was found to be distributed in 10 genera and 106 species in the current investigation. The most predominant viral species were *Staphylococcus* phage SPbeta-like, *Bacillus* virus SPbeta, *Choristoneura fumiferana* granulovirus, *Lactobacillus* phage Sha1, *Lactobacillus* phage LF1, *Lactobacillus* phage phiAT3, Oxbow orthohantavirus, BeAn 58,058 virus, *Arthrobacter* phage Mufasa8 and *Staphylococcus* virus Andhra (Fig. 7).

## Discussion

The tribal communities possess qualities and characteristics that make it a unique cultural, social, and political entity. Food and beverage making is an integral part of the culture of many of such societies. There are growing attempts worldwide to understand and document the structure and metabolic potential of the microbial community associated with ethnic fermented foods and beverages^13^. In the present study, we have investigated the microbial diversity and metabolic potential of a starter culture called *keem,* used for making various ethnic alcoholic beverages by Jaunsari tribes in the state of Uttarakhand, India. Previously, 16S rRNA/ ITS region amplicon sequencing has been used by various researchers for the identification and characterization of microbial communities associated with the starter cultures used to prepare ethnic beverages in different parts of the world^5,6,14,15,18^. We have, however used whole genome shotgun sequencing on the Illumina platform to define the microbial ecology as well as metabolic potential in the test inoculum. Whole genome sequencing is a recent approach to define microbial community composition as well as metabolic pathways in an environmental sample. The technique has been applied to understand the microbiome of traditional starter cultures also^6,15^. Shah et al. (2019)^12^ detected antagonistic properties against specific human pathogens in traditionally prepared starter inoculums by certain ethnic groups, where yeast, filamentous fungi/molds, and lactic acid bacteria coexisted.

In the present investigation, the overall analysis of the microbial community structure of *keem* revealed an alpha diversity of 123 species. Previously, Bora et al. (2016)^6^ have reported an alpha diversity of 68.84 species in a traditional rice wine starter culture *xaj-pitha* prepared by the Ahom community of Assam, India. Among the microbial community of *keem,* bacteria were the most abundant domain comprising 95.81% of total analyzed sequences followed by fungi, archaea, and viruses. On the other hand, in most of the previously published reports, fungi were reported as the most abundant domain in different traditional starter cultures used to prepare alcoholic beverages^5,6,15,19^. In our study, Actinobacteria, Firmicutes and Proteobacteria were found as the most abundant phyla among bacteria. In similar studies carried out by previous workers to define bacterial communities of different starter cultures, Proteobacteria and Firmicutes were found predominant in *xaj-pitha*^6^, Firmicutes in *emao*^15^; Proteobacteria, Actinobacteria and Firmicutes in *thiat*^14^; Proteobacteria and Firmicutes in *marcha*^1^. The dominance of Actinobacteria in *keem* may be due to the use of local medicinal herbs providing a suitable environment for the growth of this group. Bacteria belonging to this group are well known for their ability to produce secondary metabolites such as vitamins, enzymes, and antibiotics. The multifaceted abilities of Actinobacteria to produce bioactive compounds, influence flavor, improve texture, and contribute to preservation make them essential components of various fermentation processes used in food, beverage, and biotechnology industries^12^.Two-thirds of all the antibiotics known and used so far in pharmaceuticals have been obtained from actinobacteria only^20^. Among fungi, Ascomycota was recorded as the most abundant phyla in our findings. The ascomycetes are cosmopolitan in distribution and have great importance to humans for their ability to produce alcoholic beverages, bread and medicinally important compounds such as organic acids, enzymes, antibiotics, etc.^21^ Similar findings have been reported by Bora et al. (2016)^6^ and Sha et al. (2017)^14^ while studying the starter cultures of the traditional beverages *xaj-pitha, thiat* and *marcha*. Narzary et al. (2021)^15^ have, however, found Mucoromycota as the largest fungal community in the starter culture *emao.* Taxonomic classification of the bacterial population of *keem* at the genera level led to the identification of *Saccharopolyspora* as the most abundant genus followed by *Brevibacterium* and *Streptomyces*. Bacteria belonging to the genus of *Saccharopolyspora* are well known for their potential to produce a variety of industrially relevant enzymes (xylanase, amylase, protease, etc.), antibiotics, flavoring compounds and other bioactive metabolites with potential pharmaceutical or therapeutic applications^22^. Members of the genus *Brevibacterium* are widely recognized for producing amino acids, aromas, carotenoids, and antimicrobial substances^23^. *Streptomyces* is one of the most important bacterial genera that have an extraordinary capacity to bioconvert different substrates into products of industrial importance. Members of this group are widely distributed in nature and produce a wide array of secondary metabolites such as antibiotics, antifungal, antiviral, immunosuppressants, and antitumor agents having immense biomedical applications^24^. The findings thus prove that *keem* is a reservoir of several useful bacteria that may have a plethora of biotechnological applications.

Lactic acid bacteria (LAB) play an important role in the fermentation of cereal-based substrates to prepare alcoholic beverages. Although recognized as a spoiling agent if present in higher amounts, this group of bacteria provides good taste and quality as well as maintains the low pH of alcoholic beverages^25–27^. The LAB species are also considered as good producers of bacteriocins, a potential alternative to traditional antibiotics^15^. They also provide acidic conditions during the fermentation process which favors the growth of amylolytic yeast and fungi and inhibits the growth of unwanted harmful bacteria^28^. The most common LAB species reported to be present in various starter cultures so far include *W. cibaria, Weissella confuse, W. paramesenteroides, P. pentosaceus, L. mesenteroides, L. citreum*^15^. In the present study, the LAB population of *keem* was found to belong to 6 different genera, and members of each one of them have been previously reported to be present in one or other study undertaken on different starter cultures^15^. At the species level, however, we have detected 3 lactic acid bacteria namely *L. suionicum, L. johnsonii* and *L. reuteri* which have not been reported to be present so far in any of the starter inoculum used for traditional alcohol fermentation. *L. suionicum* is a heterofermentative LAB species involved in the fermentation of a range of fermented foods & beverages. Because of its metabolic abilities, it offers potential health benefits and holds importance for biotechnology industries^29^. *L. johnsonii and L. reuteri* are good probiotic agents and are also known to exhibit extensive antimicrobial activities against various human pathogens^13,30^.

The yeast community present in a starter culture is important for its ability to produce alcohol through the catabolism of carbohydrates present in a substrate. *S. cerevisiae, P. kudriavzevii, Candida orthopsilosis, Candida glabrata, D. hansenii, K. marxianus* and *T. delbrueckii* are some common yeast species identified in starter cultures used to prepare traditional alcoholic beverages^15^. In addition to that, *Kluyveromyces lactis, Candida albicans, E. gossypii, Eremothecium cymbalariae, Eremothecium sinecaudum, K. Africana, L. thermotolerans, N. dairenensis, L. thermotolerans* and *Z. rouxii*, although not so common, have also been reported to be present in ethnic inoculum^15^. We have found both common (*P. kudriavzevii*, *S. cerevisae, C. orthopsilosis, C. glabrata D. hansenii*, *K. marxianus* and *T. delbrueckii*) and uncommon (*C. albicans*, *L. thermotolerans*, *N. dairenensis, Eremothecium* sp.) yeast species in *keem*. Mycelial fungi are also known to be present in the starter culture for alcohol fermentation where they may be associated with antibacterial, amylolytic, ethanol fermenting and anti-oxidant producing abilities^31^. In our study we have detected *A. oryzae, A. fumigatus, T. terrestris, C. higginsianum, Talaromyces rugulosus, N. crassa* and *T. thermophilus* as the predominant type of molds present in *keem*. *Aspergullus oryzae* is known to secrete extracellular enzymes such as amylases including alpha-amylase, which accelerate the degradation of cereals or grains and provide more nutrients for microbes^32^. Boro and Narzary, 2022^31^ have isolated and identified various molds such as *Mucor circinelloides, Rhodosporidiobolus ruineniae, Mucor indicus, Penicillium citrinum* in the ethnic beer starter *emao* used by the Bodo community of Assam, India.

The shotgun sequence reads can be mapped to databases of orthologous gene groups such as COG^33^, KEGG^34^, NOG^35^,etc., to recognize the base-pair matches to proteins or genes with known and annotated functions. This predicts the biological function of the identified homologous gene instead of the taxonomic identity. While using KO and COG annotation in our study we found that the maximum sequences i.e., 66.73 and 50.44%, respectively, were assigned for metabolism. On the other hand, a very high proportion of the sequences i.e., 75.28%, remained poorly characterized through NOG annotations. In our analysis, the subsystem annotation revealed a maximum number of genes belonging to carbohydrates followed by clustering-based subsystems; amino acids and derivatives; cofactors, vitamins, prosthetic groups, pigments; fatty acids, lipids, and isoprenoids; protein metabolism; RNA metabolism; DNA metabolism. The presence of genes responsible for carbohydrate metabolism in *keem* determines its potential role in utilizing carbohydrate-rich raw materials for alcohol fermentation. Very little information is available about the use of different annotations for predicting metabolic potential from the whole genome sequence information of traditional starter culture. Bora et. al. (2016)^6^ found 34.9 and 46.9% of whole genome sequences of *xaj-pitha* belonging to metabolism under COGs and KO annotations. The predictive gene analysis showed that various genes responsible for different metabolic interactions are associated with the microbiome of *keem*. In the present investigation, the metabolic pathways of amino acid biosynthesis were found to harbor the maximum number of genes in a statistically significant manner through KEGG annotation within the metabolism category. Pathways such as alanine, aspartate, and glutamate metabolism, as well as biosynthesis, play a crucial role in enhancing flavor and aroma compounds during the fermentation of traditional alcoholic beverages^36^. Additionally, genes involved in the biosynthesis of branched-chain amino acids like valine, leucine, and isoleucine were also identified through metagenome analysis of *keem*. These genes could potentially contribute to the energy metabolism of lactic acid bacterial species^37^. Much similar to our study Das et al. (2023)^38^ have reported amino acid metabolism as one of the predominant predictive functional super-pathways within the metabolism category while studying metagenomics and their predictive functional analysis of fermented bamboo shoot food of Tripura, North East India. Thus, based on the results of these annotations the microbial population of *keem* appears to be comparatively metabolically more active and diverse.

## Conclusion

This current study breaks new ground in describing the community structure and potential metabolic capabilities of microorganisms associated with the traditional starter inoculum *keem*. Taxonomic and functional hit distribution were performed using MG-RAST. The analysis revealed that bacteria were the predominant microorganisms in the *keem* sample, followed by Eukaryota and archaea. The bacterial population encompassed various phyla, with the most abundant being actinobacteria, firmicutes, and proteobacteria. The bacterial population was characterized down to the species level, identifying several LAB species not previously reported in traditional starter inoculums. Various yeast and fungal species were also identified at the species level in the test sample. When comparing the microbial community of *keem* with other traditional starter cultures, qualitative and quantitative differences were observed. These differences might arise from variations in the raw materials used, preparation and preservation methods, and incubation periods. In the present investigation, sequence-based annotations unveiled the highest number of genes related to metabolism, suggesting the involvement of diverse enzymes with various functions. The microbial population of *keem* can thus be exploited in the future to isolate various enzymatic genes with potential applications, using both culture-dependent and culture-independent approaches. Furthermore, the potential use of *keem* as an inoculum for industrial fermentation could be explored in the future.

## Materials and Methods

### Sample collection

Five representative samples of traditionally prepared fresh *keem* cakes were collected from different villages of the Jaunsar-Bawar tribal region (Table 1), Uttarakhand during October, 2020.

**Table 1:** Details of sampling sites.

Sampling sites		Coordinates		
Village name	District/ state	Latitude	Longitude	Altitude (m asl)
Bayala	Dehradun/Uttarakhand	30°45′10.31″ N	77°46′3.74″ E	2175
Lohari	Dehradun/Uttarakhand	30°45′2.98″ N	77°48′46.66″ E	2328
Dasau	Dehradun/Uttarakhand	30°42′26.91″ N	77°47′36.39″ E	1750
Sakrol	Dehradun/Uttarakhand	30°33′23.06″ N	77°55′12.87″ E	1038
Lachha	Dehradun/Uttarakhand	30°33′31.53″ N	77°58′11.49″ E	1387

### Genomic DNA extraction

The *keem* samples were mixed by taking 5 g of each in a mortar pestle. 1 g of this composite mixture was used for the extraction of total genomic DNA by using the method as described previously ^39,40^. For this, 300 µl SDS solution (20% SDS dissolved in 100 mM Tris HCl, pH 8.0), glass beads (0.3 g; diameter 0.1 mm), and 500 µl TE (T_10_E_1_) saturated phenol solution were added to the suspension, and the mixture was vortexed vigorously in a homogenizer for 30 s. This was followed by centrifugation at 20,000 g for 5 min at 4 °C. Thereafter, phenol–chloroform extraction was performed, and 250 µl of supernatant was taken for isopropanol precipitation. Metagenomic DNA was quantified spectrophotometrically (Qubit Fluorimeter, Thermo Fischer, USA) and 400 ng metagenomic DNA was suspended in 200 µl T10E1 buffer and stored at − 20 °C for library preparation.

### Whole metagenome shotgun sequencing

For whole genome shotgun sequencing, the samples were outsourced to Theomics Private Limited, Bangalore, India. The whole metagenome sequencing library was constructed using the KAPA Hyper Prep Kit (KAPA Biosystems, USA) and 150 bp paired-end reads were generated on a Novoseq 6000 (Illumina Inc, USA). A total of 60 million paired-end reads were targeted as output. By using bcl2fastq (version 2.19) the sequence reads were converted to FASTQ format for further analysis.

### Quality control of sequencing reads

A quality control was first performed on the sequence reads in which low-quality bases were trimmed. In addition to that human and plant genome reads were identified, masked and duplicated reads were removed. Trimmomatic (version 0.33, parameters: ILLUMINACLIP:TruSeq3-PE-2.fa:2:30:10:8:true LEADING:20 TRAILING:20 SLIDINGWINDOW:3:15 MINLEN:60) tool was used to trim the raw reads to clip Illumina adapters and remove low-quality bases at both ends. Reads that were less than 60 bp in length were discarded after trimming. Lastly, the duplicate reads were removed by using PRINSEQ-lite (version 0.20.4, parameters: -derep 1). Paired reads that were not assigned to any human or plant genomes were only considered for downstream analysis.

### Function prediction and abundance determination

The methodology as mentioned by Kishikawa et al. (2020)^41^ was used for functional annotation and abundance detection in *keem* metagenome in the present study. For this, MEGAHIT (version 1.1.2, parameters: –min-contig-len 135) was used for assembling filtered paired-end reads into contigs. For the prediction of Open Reading Frames (ORFs) present on the contigs, the gene finder tool MetageneMark (version 3.38, parameters: -a -k -f G). CD-HIT (version 4.7, parameters: -n 5 -c 0.95 -G 1 -aL 0.9 -aS 0.9 -g 1) was used for constructing a non-redundant ORF catalog that contained 42,581,555 microbial ORFs. Thereafter, the ORF catalog was annotated with two protein databases, UniRef90 and KEGG. In the UniRef90 database, prokaryotic, viral, and fungal data was selected. For KEGG genes, a database of prokaryote KEGG genes and MGENES was utilized. The putative amino acid sequences translated from the ORF catalog were aligned against both databases with DIAMOND using BLASTP (v0.9.4.105, parameters: f 6 -b 15.0–k 1 -e 1e-6 –subject-cover 50). For quantification of ORF abundance, we mapped the filtered paired-end reads to the assembled contigs using bowtie2 with default parameters (version 2.3.2). To avoid the bias of the gene size, the ORF abundance was defined as the depth of each ORF’s region of the ORF catalog according to the mapping result. Kraken and Krona Plots were used to represent the microbial diversity abundance using Pavian R package v1.0.0 whereas MG-RAST server was also used to perform functional metagenome analysis of *keem* metagenome.

### Supplementary Information

Supplementary Figure S1.

Supplementary Table S1.

Supplementary Table S2.

## Data Availability

The datasets used and/or analysed during the current study have been submitted to NCBI Sequence Read Achieve (SRA) under the accession number PRJNA1005822.
