# Supplementary material for: Unveiling the microbial communities and metabolic pathways of Keem, a traditional starter culture, through whole-genome sequencing
Source: Sci Rep. 2024 Feb 18;14:4031. doi: 10.1038/s41598-024-53350-3 (PMC10874962; doi:10.1038/s41598-024-53350-3)

**Microbial community structure and metabolic pathway analysis of *Keem* (a starter culture) native to Jaunsari tribe of Uttarakhand, India**

Babita Rana^1^, Renu Chandola^1^, Pankaj Sanwal^2^ & Gopal Krishna Joshi^1*^

^1^Department of Biotechnology, School of Life Sciences, Hemvati Nandan Bahuguna Garhwal University, Srinagar Garhwal, Uttarakhand, India.

^2^Department of Biochemical Engineering, BTKIT, Dwarahat, Uttarakhand, India.

*Corresponding author, email: [gkjoshi@rediffmail.com](mailto:gkjoshi@rediffmail.com)

Supplementary Data Figure S1: The rarefaction curve of annotated species richness of *Keem* metagenome.


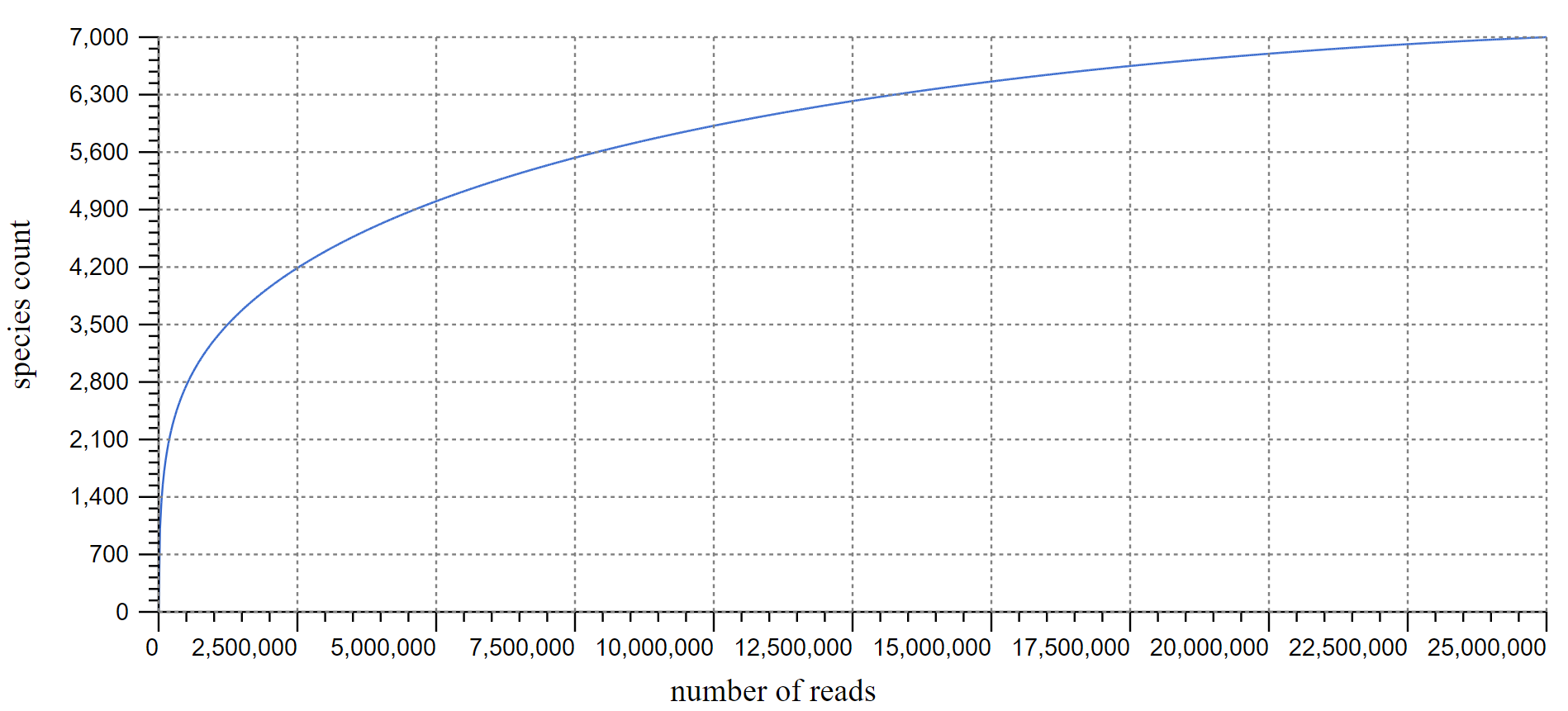

Supplement: Supplementary file 1 — Supplementary Figure S1. [file 41598_2024_53350_MOESM1_ESM.docx]
