# Supplementary material for: Unveiling the microbial communities and metabolic pathways of Keem, a traditional starter culture, through whole-genome sequencing
Source: Sci Rep. 2024 Feb 18;14:4031. doi: 10.1038/s41598-024-53350-3 (PMC10874962; doi:10.1038/s41598-024-53350-3)
Supplement: Supplementary file 2 — Supplementary Table S1. [file 41598_2024_53350_MOESM2_ESM.docx]

**Microbial community structure and metabolic pathway analysis of *Keem* (a starter culture) native to Jaunsari tribe of Uttarakhand, India**

Babita Rana^1^, Renu Chandola^1^, Pankaj Sanwal^2^ & Gopal Krishna Joshi^1*^

^1^Department of Biotechnology, School of Life Sciences, Hemvati Nandan Bahuguna Garhwal University, Srinagar Garhwal, Uttarakhand, India.

^2^Department of Biochemical Engineering, BTKIT, Dwarahat, Uttarakhand, India.

*Corresponding author, email: gkjoshi@rediffmail.com

Supplementary Data Table S1: Description of sampling sites and statistical analysis of the whole genome sequencing of *Keem*.

| **S. No.** | **Parameter** | **Value** |
| --- | --- | --- |
| 1. | Target community | Jaunsari tribal community, Uttarakhand, India |
| 2. | Date of sampling | October month, 2020 |
| 3. | GPS locations of the sample collection sites | Site1: 30°45'10.31"N; 77°46'3.74"E  Site2: 30°45'2.98"N; 77°48'46.66"E  Site3: 30°42'26.91"N; 77°47'36.39"E  Site4: 30°33'23.06"N; 77°55'12.87"E  Site5: 30°33'31.53"N; 77°58'11.49"E |
| 4. | Sample processed for whole genome sequencing | *Keem* collected from the collection sites |
| 5. | bp Count | 3,374,991,940 bp |
| 6. | Sequences Count | 22,350,940 |
| 7. | Mean Sequence Length | 151 ± 0 bp |
| 8. | Mean GC percent | 62 ± 12 % |
| 9. | Artificial Duplicate Reads: Sequence Count | 13,178,655 |
| 10. | Post QC: bp Count | 1,156,651,659 bp |
| 11. | Post QC: Sequences Count | 8,665,213 |
| 12. | Post QC: Mean Sequence Length | 133 ± 25 bp |
| 13. | Post QC: Mean GC percent | 63 ± 13 % |
| 14. | Processed: Predicted Protein Features | 4,227,668 |
| 15. | Processed: Predicted rRNA Features | 28,414 |
| 16. | Alignment: Identified Protein Features | 1,602,147 |
| 17. | Alignment: Identified rRNA Features | 8,199 |
| 18. | Annotation: Identified Functional Categories | Undefined |
